# Pathogenesis of Disease1Read before the annual meeting of the Pennsylvania State Veterinary Medical Association at Philadelphia, March, 1896.

**Published:** 1896-08

**Authors:** W. L. Rhoads

**Affiliations:** Lansdowne, Pa.


### PATHOGENESIS OF DISEASE.[1]

By W. L. RHOADS, D.V.S.,
LANSDOWNE, PA.

During yesterday's meeting I heard some of our friends say that they thought we should endeavor to keep up with the times. To do this we have determined the veterinary profession shall become the foundation upon which the science of protecting the public health will depend. We can best accomplish this object by a thorough and comprehensive study of the origin and development of disease or any departure from the state of health, either functional or organic. While the physician attempts to regulate and control the disease after it has found lodgement in our systems, the veterinarian strives to prevent its introduction into our bodies by purifying the meat- and milk-supply of the community, as they are probably the most potent factors in the development and transmission of disease. Meat left uncovered in the open air for a few hours in a moderate temperature soon becomes thoroughly infested with bacteria, especially if moisture is present. Milk is a most favorable medium for the nourishment and development of the germs of disease. These germs may come from the cow, as is the case with tuberculosis and parenchymatous mastitis, which have produced throughout a milkman's route gastro-intestinal catarrh; or the germ may be added to the milk from human sources, as in the case of scarlet fever, diphtheria, or typhoid fever. Every epidemic of typhoid in civilized and enlightened countries is now traced to infection from milk, or water and milk have proved to be the better media for its transmission. As a considerable percentage of the milch cows in a civilized country are consumptive, and as germs of this dread disease are transmitted through the milk from such cows to human consumers, it becomes necessary for enlightened and progressive boards of health to assume the sanitary supervision (through their veterinarian) of the milk-supplies of their respective communities.

I realize I have selected a subject far beyond the limit of my brief experience, and those practitioners who fail to keep abreast with the current veterinary and medical literature are losing much valuable information on this topic of most vital impor-

[1] Read before the annual meeting of the Pennsylvania State Veterinary Medical Association at Philadelphia, March, 1896.

tance. I feel no question of medicine in late years has occupied so much space and thought, or caused so much scientific research and experiment, as the origin, development, and communication of disease. We to-day have a knowledge, though yet limited, of organisms infinitely small, the existence of which was not suspected by the ordinary practitioner till within the last few years. When we consider that some of these germs when mature are but seven one-thousandths of an inch in length and their breadth scarcely one-fifth their length, our ignorance of them in the past is not surprising. We now feel that we understand fairly well the relation such organisms bear to our own as well as the organisms with which we have to deal in the lower animals in health and disease. It is true many of the lower organisms, both animal and vegetable, have for years been recognized as the direct cause of local maladies, both external and internal, for many of these parasites were of sufficient size to be in the aggregate seen by the naked eye or a low-power lens. When we realize that many of the most potent germs do not exceed $\frac{1}{1000}$ mm. in diameter, these bodies, so light and small, are easily influenced by movements of wind, rain, or change of temperature, coupled with the fact that no so-called case of spontaneous generation has withstood rigid investigation, we have a faint idea of the great good done by Bonnet, who between 1740 and 1793 advanced the theory of universal dissemination of seeds and spores, known as the pre-existing germ-theory (this is the oldest and probably the most widely accepted theory ever known in the history of medical science). During the same period Needham came to the conclusion that infusion of organic matter placed in hermetically closed vessels, after boiling, brought forth minute organisms; this is known as the vegetative or producing-force theory. The method of study introduced by Needham forms the basis of all subsequent experiments on this question as well as the culture researches in connection with the organisms of disease. Dr. Budd, in 1849, claimed the body was invaded by organisms which, under certain conditions and surroundings, caused infectious diseases. It is only of late years, in fact, since 1870, that we have been shown the relation of micro-organisms to those diseases which most closely interest the veterinarian, as anthrax, septicæmia, cholera in its various forms, lupus, glanders, rabies, pleuro-pneumonia, favus, and the greatest of all scourges, tuberculosis. Koch and Pasteur have done much in later years to enlighten us upon this subject.

The satisfying of all the requirements essential to prove a germ causative of a disease is difficult and has led to what may be termed a special branch of medical art.

Brefalo has observed that bacteria may divide once every half-hour, and their progeny repeat the process in the same time, thus producing in twenty-four hours segments amounting to many million spores, or resting cells; these are the smallest segments or cocci into which the filament at length breaks up. Yet it is questionable whether the bacteria suspended in the air are so plentiful as was once supposed. Formerly the great difficulty seemed to lie in distinguishing accurately between a germ and spore, the words being used in an indefinite sense. A *germ* is the first principle of anything that has life, whether animal or vegetable, a growing point, the embryo of a germinating seed, the exact point from which the life and organization of the future plant are to spring. A spore is a fecundated seed of a lower organism, and, though it may be practically considered the originator of a disease, in a true sense it cannot be, for the elements of which it is composed derive some consideration. Yet if the pre-existing spore is not present, it then becomes most difficult to account for the minute infusoria found swarming fully developed in putrefying fluid or flesh; in themselves scientifically healthy, though not rendered antiseptic, though they may be placed in antiseptic surroundings. It has been frequently shown that pure cultures of fungi grown on gelatin decompose, and the fluid in which they are soon swarms with bacteria.

The fungi being so minute, we have little true knowledge of them, and the infusoria may come from fungi through development by heterogenesis. Would this not lead us into the belief that the light-giving forces are in themselves spontaneous generators of disease from germs pre-existing? We are told to-day by scientists that light is a most potent factor in the destruction of ptomaïns. Prof. Ward, of England, goes so far as to assert, after experimenting with the electric light, that the light-rays and not the heat-rays are destructive to bacteria, and that of the different colored rays the blue rays are the most effective for this purpose, while another scientist claims the yellow or orange rays to be most conducive to the propagation of bacteria.

Anthrax was formerly attributed to a minute microscopic fly, which, on account of their great number, formed a blue mist in the atmosphere. This was the first disease to have conclusive proof offered of its germ origin. The question of its etiological relation was placed on a scientific basis by inoculation with the pure culture of the anthrax bacillus.

To Pasteur belongs the credit for experiment or preventive inoculation with his attenuated virus in many diseases. Animals inoculated with the cultivated bacillus show immunity from disease when reinoculated with the deadly wild germ. The cause for this has not as yet been determined.

Metschnikoff, in 1884, advanced the theory that the white corpuscles eliminated the bacilli from the blood. Germs, as a rule, are very tenacious of life, and, while light is now believed to destroy them, it has only been a short time since scientists told us that boiling would destroy them, yet they are known to have withstood as low as 100° C. and as high as 110° C., the ripe spores showing the greater powers of resistance. They flourish best in moisture at a temperature of about 35° C., and readily live on remains of dead organisms, or, truly parasitical, being nourished direct from a living body. As a rule, their ends are rounded, and they frequently present a beaded appearance if not isolated; though their length may vary, they are always evenly proportioned, and I trust the future will give such magnifying powers that we may be enabled to see that each of these spores is an organism within itself, though communication with its neighbor may be essential to its propagation or development.

We are striving to learn the why and the wherefore, the cause and effect, and, with a proper knowledge of these, diseases may be treated intelligently, practically, and successfully. It is for the accomplishment of this end that animals, from the tiny mouse to the spiteful terrier, have been subjected to injections of different brands of germ-life, and while they may be dismissed slightly damaged or sent to their last reward, the slight inconvenience is not to be compared with the knowledge gained. The veterinarian to be abreast with the times must have in his medicine-chest an antitoxin for pneumonia, tetanus, tuberculosis, rabies, and many kindred diseases.

Disease is largely a problem of political economy, and is influenced by the ebb and flow of human affairs. An eve of prosperity greatly reduces disease; the people more readily recognize the difference between good and bad food, an adequate amount of rest, and more perfect sanitation. When the masses have become thoroughly aroused and realize these to be essentials to health and longevity, then disease will become a brief, infrequent reparative process.

No living germ or disease can resist the antiseptic power of essence of cinnamon for more than a few hours is the conclusion announced by M. Chamberland as the result of prolonged research, and in M. Pasteur's laboratory it is said to destroy microbes as effectually as corrosive sublimate.

**On to Buffalo!**

